# Overhead photoselective shade films mitigate effects of climate change by arresting flavonoid and aroma composition degradation in wine

**DOI:** 10.3389/fpls.2023.1085939

**Published:** 2023-01-27

**Authors:** Lauren E. Marigliano, Runze Yu, Nazareth Torres, Cristina Medina-Plaza, Anita Oberholster, Sahap Kaan Kurtural

**Affiliations:** Department of Viticulture and Enology University of California, Davis, Davis, CA, United States

**Keywords:** anthocyanins, C13-norisoprenoids, climate change, esters, flavonols, ketones

## Abstract

**Introduction:**

Overhead photoselective shade films installed in vineyards improve berry composition in hot grape-growing regions. The aim of the study was to evaluate the flavonoid and aroma profiles and composition of wines from Cabernet Sauvignon grapes (Vitis vinifera L.) treated with partial solar radiation exclusion.

**Methods:**

Experimental design consisted in a randomized experiment with four shade films (D1, D3, D4, D5) with differing solar radiation spectra transmittance and compared to an uncovered control (C0) performed over two seasons (2021 and 2022) in Oakville (CA, USA). Berries were collected by hand at harvest and individual vinifications for each treatment and season were conducted in triplicates. Then, wine chemical composition, flavonoid and aromatic profiles were analyzed.

**Results:**

The wines from D4 treatment had greater color intensity and total phenolic index due to co-pigmentation with anthocyanins. Shade film wines D5 and D1 from the 2020 vintage demonstrated increased total anthocyanins in the hotter of the two experimental years. In 2021, reduced cluster temperatures optimized total anthocyanins in D4 wines. Reduced cluster temperatures modulated anthocyanin acylation, methylation and hydroxylation in shade film wines. Volatile aroma composition was analyzed using gas chromatography mass spectroscopy (GCMS) and D4 wines exhibited a more fruity and pleasant aroma profile than C0 wines.

**Discussion:**

Results provided evidence that partial solar radiation exclusion in the vineyard using overhead shade films directly improved flavonoid and aroma profiles of resultant wines under hot vintage conditions, providing a tool for combatting air temperatures and warmer growing conditions associated with climate change.

## 1 Introduction

It has been long recognized that the quality of wines is closely associated with the accumulation of secondary metabolites, specifically flavonoids and volatile organic compounds that have a direct effect on wine color, taste and aroma (Cortell and Kennedy, 2006; Torres et al., 2020). Flavonoids in wine include anthocyanins, flavonols and flavan-3-ols. Wine color, particularly its hue and intensity, are strongly determined by anthocyanin methylation, acetylation, hydroxylation of the anthocyanin B-ring, and co-pigmentation with cofactors such as flavonols (He et al., 2012a; Savoi et al., 2020).

Partial solar radiation exclusion was shown to effect anthocyanin hydroxylation. Tarara et al. (2008) demonstrated increased dihydroxylation of anthocyanins in grape berries exposed to direct solar radiation compared to shaded fruit. Likewise, Martínez-Lüscher et al. (2017) monitored anthocyanin hydroxylation under colored photoselective shade nets and found that by reducing solar radiation by 40% with black polyethylene shade nets, the ratio of tri- to di-hydroxylated anthocyanins was increased compared to uncovered control fruit. Such shifts in anthocyanin hydroxylation can impact anthocyanin hue and wine antioxidant capacity (Muñoz et al., 2009).

Wine aroma in both red and white wines is a matrix formed by a variety of volatile compounds. However, the composition of the matrix can be impacted by grape cultivar, vineyard conditions and fermentation conditions. Contribution of volatiles to wine flavor composition is related to its chemical structure (Zhang et al., 2021). The most abundant class of volatile compounds found in the wine matrix are higher alcohols (Zhang et al., 2021). These by-products of yeast nitrogen metabolism are usually described by unpleasant “solvent” or “fusel” aromas when present in concentrations greater than 400 mg/L (Ferreira et al., 2000; Zhang et al., 2020). The more pleasant “fruity” aromas described in wine are associated with esters. Esters are often in highest concentrations in young red wines and decrease in concentration with aging (Câmara et al., 2006). C13-norisoprenoids and terpenes are key aromas compounds found in both red and white wines, contributing fruity and floral aromas at low olfactory concentrations (Rapp, 1998). C13-norisopenoids are understood to be derivatives of enzymatic or photochemical degradation of carotenoid pigments in the grape berry (Isoe et al., 1972). In plants, carotenoids have photo-protectant and antioxidant properties, making these pigments responsive to solar radiation in grape berries. Carotenoids in grape berries have been shown to increase in berries with increased in solar radiation pre-veraison (Marais et al., 1992; Gerdes et al., 2002). However, under extreme exposure to heat and solar radiation, there is a documented decrease in carotenoid concentrations during ripening (Oliveira et al., 2004). To preserve the carotenoid concentrations in the grape berry and to promote C13-norisoprenoids in resulting wines under more frequent heat wave events and increases in air temperature, artificial shading with black polyethylene cloth has been trialed and found that shaded fruit contained more carotenoids than unshaded fruit (Lu et al., 2021). However, the effect of partial solar radiation exclusion on wine C-13 norisoprenoid content seems to be more nuanced. Wines produced from the shaded fruit contained more β- damascenone as well as esters compared to wines produced from unshaded fruit (Lu et al., 2021). Yet, there are conflicting reports showing no effect of UV exposure on β- damascenone concentrations in Shiraz wines made from clusters that underwent solar radiation exposure *via* varying rates of leaf removal and polycarbonate UV screens (Song et al., 2015). Like C13-norisoprenoids, final terpene concentrations in wines depends on the net accumulation in grape clusters exposed to excessive temperatures and UV radiation (Song et al., 2015; Miao et al., 2020).

The effect of photoselective overhead shade films on whole plant physiology and temporal development of berry flavonoids of Cabernet Sauvignon development over two growing seasons was previously studied in a hot region (Marigliano et al., 2022). Grape berries growing under reduced near-infrared radiation exposure in hotter than average years, resulted in a 27% increase in anthocyanin content at harvest than the exposed control due to decreases in anthocyanin degradation due to high berry temperatures (Marigliano et al., 2022). Moreover, flavonol degradation was similarly decreased, thus optimizing flavonol content in the grape berry under reduced near-infrared radiation exposure (Marigliano et al., 2022). The objectives of this study aimed to determine the extent to which the impact of photoselective overhead shade films on flavonoid development transfer to wine and the cascading effects of partial solar radiation exclusion had on aroma composition of resultant wines.

## 2 Materials and methods

### 2.1 Chemicals

Chemicals of analytical grade included 2-undecanone. All chromatographic solvents were of high-performance liquid chromatography (HPLC) grade including acetonitrile, methanol, hydrochloric acid, and formic acid. These solvents were purchased from Thermo-Fisher Scientific (Santa Clara, CA, USA). HPLC-grade standards including quercetin 3-*O*-glucoside and malvidin chloride were purchased from Extrasynthese (Genay, France).

### 2.2 Plant material, experimental design, and overhead shade film treatments

The experiment was conducted in Oakville, CA, USA during two consecutive growing seasons (2020 and 2021) at the University of California Davis, Oakville Experimental Vineyard. The vineyard was planted with “Cabernet Sauvignon” (*Vitis vinifera* L.) clone FPS08 grafted onto 110 Richter rootstock. The grapevines were planted at 2.0 m × 2.4m (vine × row) and oriented NW to SE. The grapevines were trained to bilateral cordons, vertically shoot positioned, and pruned to 30-single bud spurs. Irrigation was applied uniformly from fruit set to harvest at 25% evapotranspiration (ET_c_) as described elsewhere (Torres et al., 2021).

**Figure 1:**
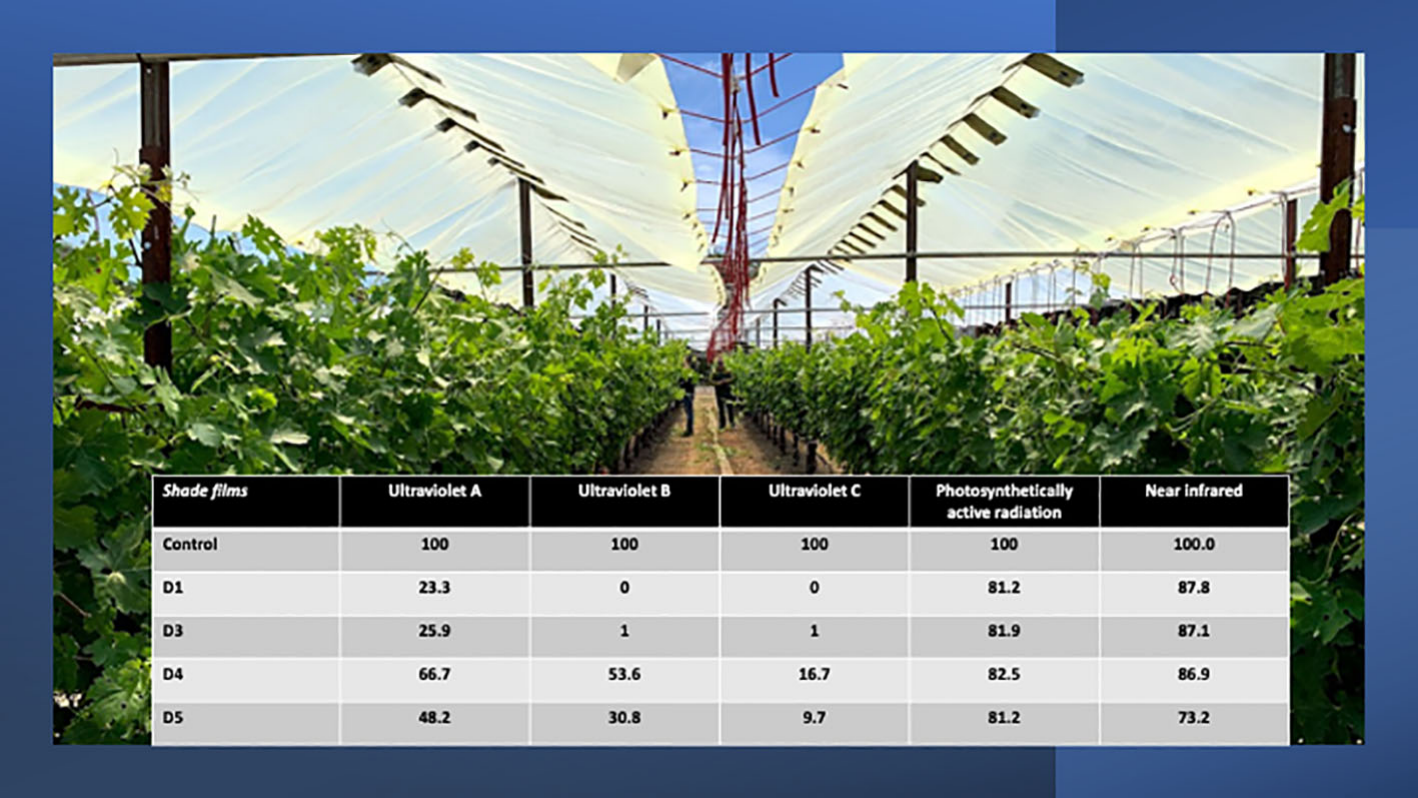
Overhead shade films installed above the experimental vineyard and the percentage of solar radiation spectra transmitted through them at solar noon. Portion of this figure previously appeared in Marigliano et al. (2022).

The experiment was arranged in a randomized complete block with four replications. The photoselective shade film treatments were previously described in Marigliano et al. (2022) and their properties presented in **Figure 1**. Shade films were designed to target portions of the electromagnetic spectrum previously observed and measured at the experimental site (Martínez-Lüscher et al., 2017; Martínez-Lüscher et al., 2019). Briefly, four photoselective shade films (Daios S.a. Naousa, Greece) and an untreated control were installed in 3 adjacent rows on 12 September 2019. The shade films remained suspended over the vineyard until 20 October 2021. The shade films were 2 m wide and 11m long and were secured on trellising approximately 2.5 m above the vineyard floor. Each experimental unit consisted of 15 grapevines in 3 adjacent rows. Grape clusters were harvested by hand from each experimental unit when berry total soluble solids (TSS) reached 25^o^Brix on 9 September 2020, and 7 September 2021, respectively.

### 2.3 Winemaking protocol

Vinification was conducted in 2020 and 2021 at the UC Davis Teaching and Research Winery. Upon arrival at the winery, grapes were destemmed and crushed mechanically. Must from each field experimental unit was divided into three technical fermentation replicates (200L each). K_2_S_2_O_2_ was added to each treatment- replicate (50 mg L^-1^ SO_2_) and must was allowed to cold-soak overnight at 5^o^C in jacketed stainless-steel tanks controlled by an integrated fermentation control system (TJ fermenters, Cypress Semiconductor Co., San Jose, CA, USA). The following day each treatment-replicate was inoculated with EC-1118 yeast (Lallemand Lalvin®, Montreal, Canada) to initiate fermentation. Musts were fermented at 25°C and two volumes of must were pumped over twice per day by the integrated fermentation control system. During the winemaking process, TSS was monitored daily using a densitometer (DMA 35, Anton Paar USA Inc., Ashland, VA, USA) and fermentations were considered complete once residual sugar contents were less than 3 g L^-1^. Wines were then mechanically pressed using a screw-type basket press. Following pressing, wine samples were collected for flavonoid analysis. Malolactic fermentation was initiated with the addition of Viniflora® *Oenococcus oeni* (Chr. Hansen A/S, Hørsholm, Denmark). Malolactic fermentation was carried out at 20^o^C. Upon completion of MLF, free SO_2_ levels were then adjusted to 35 mg L^-1^ and wines were bottled.

### 2.4 Chemical composition of wines

A 100 mL wine sample from each technical replicate was used to determine wine pH, titratable acidity (TA) and alcohol content. The pH and TA of the wines was determined using an autotitrator (Omnis titrator, Metroohm, Switzerland). The TA was determined by neutralization with NaOH up to pH 8.2 and expressed as g/L of tartaric acid. Alcohol content in the wines was determined with an alcolyzer SP-1 m (Anton Paar, Graz, Austria) by near-infrared spectroscopy.

### 2.5 Wine spectrophotometric analysis

Using a spectrophotometer (Cary 100; Agilent, CA, USA), color intensity (CI), hue, total polyphenolic index (TPI) and % of polymeric anthocyanins was determined following procedures described by Ribéreau-Gayon, Glories, Maujean, and Dubourdieu (2000). Wine samples were diluted in water (1:100 v:v) and absorbance readings were taken at 280, 420, 520, and 620nm. The absorbance at 740 nm was subtracted from all absorbance readings to eliminate turbidity. CI was calculated as the sum of absorbance at 420, 520 and 620nm. Hue was calculated as the ratio between the absorbance at 420 and 520nm. The percentage of polymeric anthocyanins was determined *via* absorbance measurements at 520nm after anthocyanin bleaching with a sodium bisulfite solution (10mg/mL). TPI was determined by diluting wines with water (1:100) and recording absorbance at 280nm.

### 2.6 Wine flavonoid concentration and composition

Wine flavonoid composition was determined following procedures previously described (Torres et al., 2022). Briefly, wine samples collected after pressing were centrifuged at 5,000 rpm for 10 mins, filtered with PTFE membrane filters (diameter 13mm, pore size: 45 μm, Thermo Fisher Scientific, San Jose, CA) and transferred to high performance liquid chromatography (HPLC) vials prior to injection. An Agilent 1260 series HPLC system with a reversed-phase C18 column (LiChrosphere 100 RP-18, 4 x 520 mm^2^, 5μm particle size, Agilent Technologies, Santa Clara, CA, USA) was used to simultaneously determine the anthocyanin and flavonol concentrations. The mobile phase flow rate was 0.5 mL min^-1^, and two mobile phases were used, which included solvent A = 5.5% aqueous formic acid; solvent B = 5.5% formic acid in acetonitrile. The HPLC flow gradient started with 91.5% A with 8.5% B, 87% A with 13% B at 25 min, 82% A with 18% B at 35 min, 62% A with 38% B at 70 mins, 50% A with 50% B at 70.01 min, 30% A with 70% B at 75 min, 91.5% A with 8.5% B from 75.01 min to 91 min. The column temperature was maintained at 25^o^C. This elution allowed for avoiding co-elution of anthocyanins and flavonols as previously reported (Martínez-Lüscher et al., 2019). Flavonols and anthocyanins were quantified by determining the peak absorbance at 365nm and 520nm, respectively. Quercetin 3-*O*-glucoside and malvidin chloride (Extrasynthese, Genay, France) were used as quantitative standards.

### 2.7 Wine aromatic profile

Volatile compounds in wine samples were analyzed following procedures described previously (Torres et al., 2022). Briefly, 10-mL of each wine sample was transferred to a 20-mL amber glass vial (Agilent Technologies, Santa Clara, CA, USA). Each vial also contained 3 g of NaCl (Sigma Aldrich, St. Louis, MO) and 50μg of an internal standard solution of 2-undecanone (10μg/L prepared in 100% ethanol). After agitating at 500 rpm for 5 mins at 30^o^C, samples were exposed to 1 cm polydimethylsiloxane/divinylbenzene/Carboxen (PDMS/DVB/CAR) (Supelco Analytical, Bellefonte, PA), 23-gauge SPME fiber for 45 mins. Helium was used as a carrier gas at a flow rate of 0.8636 mL/min in a DB-Wax 231 ETR capillary column (30m, 0.25mm, 0.25μm film thickness) (J&W Scientific, Folsom, CA, USA) with constant pressure and temperature at 5.5311 psi and 40^o^C, respectively. The oven temperature was kept at 40^o^C for 5 mins and then incrementally increased by 3^o^C/min until reaching 180^o^C. Oven temperature was then increased by 30^o^C/min until reaching 260^o^C, at which temperature was maintained for 7.67min. The SPME fiber was desorbed split mode with a 10:1 split for wine samples and held in the inlet for 10min to prevent carryover effects. The method was retention time-locked to the 2-undecanone internal standard. The total run time per sample was 61.67min. Electron ionization was performed with a source temperature of 230^o^C and the quadrupole at 150^o^C. The wine samples were measured using synchronous scan and selected ion monitoring (SIM mode). The mass spectrometer scanned from *m/z* 40 to 300. Compounds were detected using between two and six selected ions.

Data was analyzed using MassHunter Qualitative Analysis software (version B.07.00) (Agilent Technologies, Santa Clara, CA, USA). After normalization with 2-undecanone internal standard, results were expressed as peak areas. Compounds were tentatively identified in the mass spectrometry spectrum of the peaks and confirmed by comparison to the National Institute of Standards and Technology database (NIST) (https://www.nist.gov ). The ions used SIM for each compound and retention times were reported previously by (Girardello et al., 2019). The odor activity value thresholds (OAV) were obtained from a selected review of published literature of young red wines (Francis and Newton, 2005)and were used in comparing the monitored compounds (**Table S1**).

### 2.8 Statistical analysis

Statistical analyses were conducted with R Studio version 4.0.5 (RStudio: Integrated Development for R., Boston, MA, USA) for Windows. All data were subjected to the Shapiro-Wilk’s normality test. Data was subjected to two-way analysis of variance (ANOVA) to assess the statistical differences between the applied shade film vineyard treatments and the vintage and their combination. Means ± standard errors (SE) were calculated and when the *F*-value was significant (*p* ≤ 0.10), a Duncan’s new multiple range *post-hoc* test was executed using “agricolae” 1.2-8 R package (de Mendiburu, 2016). Principal component analysis (PCA) was conducted and visualized with the same software, using the “factoextra” package (Kassambara and Mundt, 2020). Pearson correlation analyses were performed with using the same software with the “corrplot” package (Wei et al., 2017).

## 3 Results

### 3.1 Experimental weather conditions

Meteorological data collection and climactic conditions at the experimental site for the 2020 and 2021 growing seasons are described in detail by Marigliano et al. (2022). Briefly, there were 1762.7°C growing degree days (GDDs) accumulated in 2020 compared to 1572.3°C GDDs accumulated in 2021, with similar GDD accumulation from April to June in both years. Compared to the 10-year average (2009-2019), the 2020 growing season accumulated more growing degree days by 1 October. The 2021 growing season was a cooler year with less accumulated GDD than the 10-year average. The total precipitation at the experimental site from 1 March 2020 to 30 September 2020 was 84.1mm, a notable 100.5mm less precipitation than the 10-year average for the experimental site. Drought conditions continued into the 2021 water year, with 66.9 mm of precipitation between 1 March 2021 and 30 September 2021. Precipitation only occurred in March and April 2021 and was negligible in the following months. Given the severe drought conditions in both experimental years, precipitation had a negligible effect on plant water status in control and shaded treatments with 25% ET_c_ replacement, as demonstrated by no significant effects on stem water potential integrals between control and shaded treatments in either experimental year (Marigliano et al., 2022).

### 3.2 Color parameters and chemical characteristics

Grapes resulting from field treatments were vinified under the same conditions in both years. In 2020 alcohol content was the highest in D1 and D4 wines (**Table 1**), while alcohol content and residual sugar concentration was lowest in C0 in 2020. All shade film wines contained more alcohol and residual sugar than C0. In 2021, alcohol content and residual sugar concentration was unaffected across all wines. In 2020, pH was only decreased in D3 wines. In 2021, C0 wines had the lowest pH compared wines from shaded grapes. Among the shaded treatments, D4 and D5 wines had higher pH compared to D1 and D3 wines. In 2020, titratable acidity only increased in D3 wines compared to C0, D1 and D5 wines. C0, D1, D4 and D5 wines were indistinguishable in titratable acidity. While C0 had one of the lowest values for TA in 2020, C0 wines in 2021 had one of the highest TA values, along with D3 and D5 wines. The lowest TA value was observed in D4 wines from 2021.

**Table 1:** Chemical and colorimetric properties of wine samples from ‘Cabernet Sauvignon’ grapevines subjected to different photoselective shade film treatments in a vineyard in Oakville, CA, USA in two growing seasons (2020 and 2021).

	2020							2021					Y	Y x S
Wine color parameters	C0	D1	D3	D4	D5	*p*-value	C0	D1	D3	D4	D5	*p*-value		
CI	12.8^a^ ±0.2c	15.1±0.9 ab	14.4±0.2bc	16.2±0.3 a	14.1±0.7bc	*	15.3±0.2 b	15.4±0.1 b	15.7±0.0 b	16.6±0.5a	15.7±0.1 b	*	***	.
hue	0.62±0.00a	0.61±0.00a	0.59±0.00b	0.63±0.01 a	0.61±0.01a	*	0.61±0.01	0.62±0.01	0.63±0.01	0.63±0.01	0.63±0.01	ns	**	*
% Polymeric anthocyanins	37.5±0.8b	38.2±0.6ab	37.0±0.6 b	41.5±1.0 a	36.2±2.0 b	.	27.9±0.4 b	28.8±0.7 ab	30.2± 0.6b	29.3± 0.3 a	29.4±0.3 b	.	***	*
TPI (AU)	47.1±1.2b	54.8± 2.9a	48.2± 0.9 b	55.1± 2.0 a	50.9±0.3ab	*	55.8± 2.6 b	64.2±1.3 ab	64.9±5.1 ab	74.4± 3.9 a	63.7±3.2 ab	*	***	ns
Chemical characteristics														
Alcohol content (%)	14.7±0.09c	15.2±0.05ab	15.2±0.03b	15.4±0.10 a	15.1±0.05b	***	15.3± 0.01	15.2 ± 0.07	15.3 ± 0.13	15.5 ± 0.17	15.3 ± 0.17	ns	**	.
Residual sugar (g/L)	0.19±0.02b	0.30±0.02a	0.26±0.01a	0.27±0.02a	0.26±0.00a	*	0.07±0.02	0.05±0.00	0.05±0.01	0.06±0.01	0.05±0.01	ns	***	**
pH	3.66±0.01a	3.64± 0.01a	3.61±0.01b	3.66±0.00a	3.66±0.00a	***	3.65±0.02c	3.69±0.01b	3.72±0.01b	3.76±0.01a	3.75±0.00 a	***	***	***
Titratable acidity (g/L)	5.45±0.04b	5.43±0.08b	5.65±0.07a	5.49±0.03ab	5.41±0.01b	.	6.12±0.01a	5.97±0.01bc	6.06±0.05ab	5.85±0.06c	6.08±0.06ab	**	***	*

a Values represent means ± (n = 3) separated by Duncan’s new multiple range post-hoc text at (α = 0.05). Means separated by different letters are significantly different within each year. AU : absorbance units, CI: Color intensity, TPI: total polyphenol index. Significance or non-significance for shade treatment, year (Y) and interactions between year and shade treatment (Y*S) are indicated by: ‘ns’= not significant; ‘.’ p≤0.1; ‘*’ p≤0.05; ‘**’ p≤ 0.01; ‘***’ p≤0.001.

Color intensity (CI) within the 2020 wines varied considerably, with the D4 having the greatest value for CI (**Table 1**). In 2021, D4 again had the highest values for CI, while the remaining wines were statistically not different from each other. Hue decreased only in D3 wines during the 2020 vintage, while there was no effect of shade films of wine hue during the 2021 vintage (**Table 1**). The trend for the percentage of polymeric anthocyanins was consistent in both vintages. D1 and D4 had the highest percentage of polymeric anthocyanins, while D5, D3 and C0 wines had less (**Table 1**). In 2020, D1 and D4 wines had higher TPI values compared to C0 and D3 wines. In 2021, TPI of wines was not affected by shade films except for D4.

### 3.3 Wine flavonoid content and profile

Wine anthocyanin profiles were separated into glucosides, 3-acetylated and coumarylated anthocyanins (**Table 2**). The total free anthocyanin concentration was the lowest in C0 wines compared to shade film treatments in 2020. Concentrations of 3-glucosides and 3-acteylated glucosides increased for all anthocyanins under shading treatments compared to C0, with the exception of peonidin 3-acetyl-glucoside and cyanidin 3-glucoside in which shading treatments had no effect. The composition of coumarylated 3’4’5’-hydroxylated anthocyanin modifications was largely impacted by shading, with the largest concentrations detected in C0, D1 and D5 wines. Overall, the ratio of di- to tri-hydroxylated anthocyanins was the largest in C0 wines and the lowest in D5. Conversely in 2021, total free anthocyanin concentrations were the highest in D4, C0, and D1 wines. Anthocyanin modifications due to shading treatments were more varied in 2021 compared to 2020. Overall, wines from D4 had the most 3-glucosides and 3-acetylated glucosides, while C0 and D5 consistently had less. Coumarylated anthocyanin concentrations were reduced in D3 and D5 wines compared to C0 wines. This was not consistent with the concentrations observed in 2020. Likewise, there was no statistically significant effect on the anthocyanin hydroxylation ratio in 2021 wines, while shading had an impact on anthocyanin hydroxylation in wines in 2020.

**Table 2:** HPLC separations of flavonoids in wines from Cabernet Sauvignon grapevines subjected to different photoselective shade film treatments in Oakville, CA, USA in 2020 and 2021 growing seasons^a^.

				2020					2021					Y	Y x S
		C0	D1	D3	D4	D5	*p*-value	C0	D1	D3	D4	D5	*p*-value		
*Anthocyanin 3-glucoside (mg/L)*	Delphinidin	2.6 ±0.1b	4.2±0.4a	3.8±0.1a	4.2±0.1a	4.1±0.1a	***	31.8±1.1bc	33.5±0.7b	31.4±1.1bc	37.2±0.4a	30.6±0.6 c	**	***	***
	Cyanidin	0.20±0.06	0.16±0.02	0.16±0.02	0.24±0.06	0.23±0.03	ns	1.1±0.0 b	1.2±0.1 b	1.3± 0.1 ab	1.5±0.2 a	1.2±0.2ab	.	***	ns
	Petunidin	3.3±0.1b	4.6 ± 0.3a	4.3 ± 0.1 a	4.5 ± 0.1 a	4.6±0.1a	**	34.4±1.7b	36.5±0.8b	34.4±1.0b	42.3±1.6a	34.1±0.4b	**	***	***
	Peonidin	1.3±0.1c	1.8±0.2b	1.7±0.04b	1.7±0.1b	2.2±0.04 a	**	10.5±0.5	9.8±0.2	11.5±1.6	14.9±4.3	9.2±0.2	ns	***	ns
	Malvidin	46.5±1.3b	53.6 ± 2.5 a	50.1 ± 1.2 ab	46.5 ± 1.2 b	53.7 ± 1.7 a	*	342.0±15.6	369.0±13.8	345.0±10.1	384.7±17.1	334.8±7.2	ns	***	ns
	Total glucosides	53.9±1.4b	64.4±3.4a	60.2±1.5ab	57.2±1.5b	64.7±2.0a	*	419.9±18.8b	450.1±15.5ab	423.6±12.2b	480.7±23.1a	409.9 ± 8.0b	.	***	*
														
*3-Acetyl-glucoside (mg/L)*	Delphinidin	0.84±0.05 b	1.41± 0.16 a	1.28±0.07a	1.46± 0.05 a	1.47 ± 0.11 a	**	11.5 ± 0.6 b	14.0 ± 0.3 ab	13.2 ± 0.5 b	17.2 ± 2.4 a	13.1 ± 0.5 b	.	***	ns
	Cyanidin	0.74± 0.05 c	0.88±0.02bc	0.94±0.03ab	0.99±0.03ab	1.08 ± 0.07 a	**	5.8 ± 0.6	6.2 ± 0.7	9.5 ± 3.0	9.7 ± 2.2	5.6 ± 1.4	ns	***	ns
	Petunidin	1.2 ± 0.1 b	2.4 ± 0.5 a	1.7 ± 0.1 ab	2.2 ± 0.3 a	2.1 ± 0.2 ab	.	13.8 ± 0.9 b	16.0 ± 0.6 ab	14.8 ± 0.6 b	18.5 ± 1.4 a	14.3 ± 0.6 b	*	***	*
	Peonidin	0.27 ± 0.03	0.20 ± 0.11	0.28 ± 0.02	0.26 ± 0.04	0.34 ± 0.03	ns	10.7 ± 0.4 ab	9.7 ± 0.3 bc	9.0 ± 0.2 c	11.6 ± 0.8 a	10.2 ± 0.5 abc	*	***	*
	Malvidin	28.5± 0.8 b	31.7 ± 1.0 a	28.7 ± 0.8 b	27.0 ± 0.6 b	31.6 ± 1.0 a	*	2.1 ± 0.2	1.9 ± 0.2	2.0 ± 0.1	2.3 ± 0.4	2.1 ± 0.2	ns	***	**
	Total acetylates	31.6± 0.9 b	36.6 ± 1.7 a	32.9 ± 0.9 b	31.9 ± 0.5 b	36.7 ± 0.8 a	*	43.9 ± 2.1 b	47.7 ± 1.9 ab	48.5 ± 3.4 ab	59.3 ± 6.3 a	45.2 ± 2.1 b	.	***	*
*3-p-Coumaroyl-glucoside (mg/L)*	Delphinidin	1.1± 0.1 ab	1.3 ± 0.05 a	1.1 ± 0.1 ab	0.94 ± 0.1 b	1.2 ± 0.1 a	*	194.4±11.0ab	212.0 ± 8.9a	189.2±6.5 ab	210.3±5.6 a	175.7 ± 4.5 b	*	***	*
	Cyanidin	0.45 ± 0.03	0.42 ± 0.1	0.37 ± 0.08	0.50 ± 0.0	0.40 ± 0.04	ns	2.7 ± 0.2 ab	2.3 ± 0.1 bc	2.1 ± 0.1 bc	3.1 ± 0.3 a	1.9 ± 0.2 c	*	***	*
	Petunidin	0.28 ± 0.01	0.39 ± 0.14	0.35 ± 0.06	0.34 ± 0.03	0.32 ± 0.03	ns	1.8 ± 0.2 a	1.0 ± 0.0 c	1.0 ± 0.2 bc	1.5 ± 0.1 ab	1.1 ± 0.1 bc	**	***	**
	Peonidin	0.28±0.01bc	0.36±0.01ab	0.27±0.05c	0.31±0.01bc	0.42±0.02a	*	3.24±0.1a	2.6±0.1b	2.6±0.2b	2.8±0.2ab	2.5±0.0b	*	***	**
	Malvidin	5.2±0.2ab	5.8±0.4a	4.8±0.05b	4.5±0.1b	5.7±0.3a	**	44.0±3.1a	43.8±2.3a	38.3±1.5ab	44.6±2.0a	35.1±1.3b	*	***	*
	Total coumarylated	6.2±0.2ab	7.0±0.5a	5.8±0.2b	5.7±0.1b	6.9±0.2a	*	51.7±3.5a	49.7±2.5a	44.0±1.7ab	52.0±2.3a	40.6±1.6b	*	***	**
	Total methylated anthocyanins (mg/L)	86.9±2.4b	100.8±5.0a	92.3±2.3ab	87.4±1.9b	100.9±2.8a	*	462.6±22.1ab	490.3±18.2ab	458.7±13.4b	523.3±26.2a	443.4±9.1b	.	***	*
	Total free anthocyanins (mg/L)	92.8±2.5b	109.1±5.6a	100.0±2.4ab	95.7±2.0b	109.4±3.0a	*	709.8±35.4ab	759.5±28.6ab	705.4±20.7b	802.3±35.9a	671.3±15.5b	.	***	*
	Ratio 3’4’5'/3'4'	36.2±3.5a	32.4±1.2ab	30.5±0.5abc	28.0±1.5bc	26.0±1.1c	*	35.3±0.1	40.0±0.4	33.0±3.8	31.3±5.8	38.2±0.7	ns	**	ns
															
*Flavonols (mg/L)*	Myricetin-3-glucoside	0.59±0.03ab	0.53±0.05b	0.51±0.01b	0.66±0.02a	0.55±0.01b	*	3.7±0.1a	2.8±0.2b	2.9±0.1b	3.4±0.1b	3.0 ±0.1b	**	***	***
	Myricetin-3-glucuronide	3.2±0.1	3.4±0.2	3.2±0.1	3.4±0.1	3.5±0.1	ns	21.4±0.5ab	18.8±0.6c	18.6±1.1c	22.4±1.1a	19.5±0.4bc	*	***	*
	Quercetin-3-galactoside	0.39±0.04ab	0.24±0.03c	0.26±0.0c	0.41±0.03a	0.31±0.03bc	**	2.0±0.1a	1.0±0.0c	1.1±0.1c	1.6±0.1b	1.2±0.2c	**	***	***
	Quercetin-3-glucoside	2.1±0.05a	1.2±0.11c	1.2±0.03c	1.6±0.03b	1.5±0.01b	***	9.8 ± 0.6a	5.8±0.1c	6.1±0.2c	7.4±0.3b	6.6±0.4bc	***	***	***
	Quercetin-3-glucuronide	2.3±0.04a	1.4± .05d	1.4±0.08d	2.0±0.08b	1.7±0.07c	***	12.0±0.8a	5.6±0.1d	6.7±0.2cd	8.4±0.0b	7.8±0.5bc	***	***	***
	Laricitrin-3-glucoside	0.98±0.03a	0.81±0.05bc	0.78±0.03c	0.91±0.03ab	0.88±0.02abc	*	3.9±0.4	3.1±0.2	3.4±0.2	4.0±0.1	3.6±0.1	ns	***	ns
	Kaempferol-3-glucoside	0.42±0.02a	0.23±0.02c	0.23±0.02c	0.31±0.01b	0.27±0.02bc	***	1.8±0.2a	0.7±0.0c	1.0±0.1bc	1.2±0.0b	1.1±0.1b	***	***	***
	Isorhamnetin-3-glucoside	0.73±0.04a	0.44±0.06b	0.38±0.01b	0.46±0.01b	0.45±0.05b	***	2.9±0.4a	1.5±0.1c	1.9±0.1bc	2.3±0.1ab	2.0±0.1bc	**	***	*
	Syringetin-3-glucoside	1.3±0.03a	1.0±0.06c	1.0±0.05c	1.2±0.02ab	1.1±0.02bc	**	4.6±0.3a	3.1±0.1c	3.5±0.0bc	4.0±0.1b	3.6± .2b	***	***	***
	Total flavonols	11.9±0.2 a	9.3±0.6 cd	9.0±0.3d	11.0±0.2ab	10.2±0.1bc	***	62.0±2.4a	42.3±0.7d	45.1±1.7cd	54.7±1.5b	48.4±1.4c	***	***	***

a Values represent means ± SE (n=3) separated by Duncan’s new multiple range post hoc test (α=0.05). Means separated by different letters are significantly different within each year. Significance or non-significance for shade treatment, year (Y) and interactions between year and shade treatment (YxS) are indicated by: ‘ns’= not significant; ‘.’ p≤0.1; ‘*’ p≤0.05; ‘**’ p≤ 0.01; ‘***’ p≤0.001.

Nine flavonol compounds were monitored in wines using HPLC (**Table 2**). For all monitored flavonol compounds except myricetin-3-glucuronide, C0 wines consistently had the highest concentrations in 2020 compared to shaded wines, with D4 and D5 wines following in flavonol concentration. Subsequently, C0 also had the highest wine flavonol concentration when calculated as total flavonols in 2020. A similar trend occurred in 2021. C0 wines from 2021 also contained greater concentrations of each flavonol compared to shaded treatments, as well as total flavonol concentration.

### 3.4 Wine aroma content and profile

The wine aroma profiles from the 2020 and 2021 vintages were analyzed with (GCMS) and 29 volatile compounds were identified and categorized into their respective compound classes (**Table 3**). The aromas profiles of wines depended highly on vintage, resulting in distinct aroma profiles. Generally, in 2020, total higher alcohols were unaffected by shade treatments, except for isoamyl alcohol and benzyl alcohol. Wines produced from shaded fruit had similar concentrations of isoamyl alcohol while the C0 had the lowest isoamyl alcohol concentration. Benzyl alcohol concentrations were reduced in D3 and D5 wines compared to C0, D1 and D4 wines. In 2021, shading treatments did not impact the concentration of higher alcohols in the resulting wines except for benzyl alcohol, which increased in 2021 D3 wines compared to all other treatments.

**Table 3:** Aromatic composition of Cabernet Sauvignon wines from grapevines subjected to different photoselective shade film treatments in Oakville, CA, USA in 2020 and 2021 growing seasons^a^.

	2020						2021						Y	Y x S
	C0	D1	D3	D4	D5	*p*-value	C0	D1	D3	D4	D5	*p*-value		
*Total C6 alcohols*														
1-Hexanol (μg/L)	18.8 ± 1.1	19.5 ± 0.9	19.2 ± 0.2	22.8 ± 2.8	18.7 ± 0.4	ns	14.1 ± 0.4 a	11.9 ± 0.5 b	11.6 ± 0.3 b	13.8 ± 0.4 a	14.4±0.4 a	**	***	ns
(*E*)-2-Hexen-1-ol (μg/L)	0.28±0.001ab	0.35±0.028a	0.27±0.017ab	0.21±0.055b	0.36±0.022a	*	0.21 ± 0.04	0.17 ± 0.05	0.17 ± 0.02	0.22 ± 0.03	0.21± 0.01	ns	***	ns
*Total higher alcohols*														
Isoamyl alcohol (mg/L)	0.84±0.04b	0.97±0.01 a	0.90±0.02ab	0.99±0.04 a	0.90±0.03ab	*	0.94±0.03	0.94 ±0.05	0.99 ±0.04	1.00 ±0.04	0.96 ±0.02	ns	*	ns
1-Octen-3-ol (μg/L)	0.75±0.03	0.66±0.3	0.67±0.2	0.96±0.2	0.83±0.09	ns	0.58±0.04	0.58±0.05	0.54±0.13	0.56±0.14	0.58±0.02	ns	*	ns
2-Phenyl-1-ethanol (mg/L)	2.1±0.1	2.3±0.1	2.1±0.1	2.3±0.1	2.2±0.1	ns	2.4±0.1d	2.4 ±0.1 c	2.8±0.2a	2.5±0.1bc	2.6±0.1ab	ns	***	ns
Isobutanol (μg/L)	0.72±0.57	1.94±0.84	0.21±0.04	1.39±0.62	1.69±0.77	ns	1.38±0.65	0.11±0.02	0.81±0.70	0.53 ±0.42	0.12±0.02	ns	ns	ns
Benzyl alcohol (μg/L)	3.1±0.08a	3.2±0.1a	2.7±0.05b	3.1±0.1a	2.9±0.1ab	*	2.9±0.1d	3.5±0.1c	4.2±0.1a	3.7±0.1bc	3.9±0.1ab	***	***	***
*Total acetate esters*														
Ethyl acetate (mg/L)	0.39±0.02c	0.47±0.00a	0.45±0.00ab	0.48±0.01a	0.42±0.02bc	**	0.36±0.01	0.35±0.02	0.37±0.02	0.38±0.02	0.36±0.03	ns	***	ns
Isoamyl acetate (mg/L)	0.31±0.02b	0.45±0.03a	0.44±0.03a	0.32±0.04b	0.37±0.01ab	*	0.49±0.01	0.47±0.07	0.43±0.04	0.46±0.03	0.44±0.04	ns	**	ns
*Total fatty acid ethyl esters*														
Ethyl hexanoate (mg/L)	1.18±0.06b	1.40±0.05a	1.25±0.06b	1.24±0.01b	1.31±0.03ab	.	0.89±0.05	0.82±0.07	0.82±0.07	0.81±0.05	0.77±0.04	ns	***	ns
Ethyl octanoate (mg/L)	9.09±0.3ab	10.5±0.6a	8.76±0.5b	9.43±0.3ab	10.2±0.2a	.	7.98±0.6	7.47±0.8	7.73±1.1	7.78±0.7	6.84±0.6	ns	***	ns
Ethyl decanoate (μg/L)	0.06±0.01bc	0.08± 0.01a	0.08± 0.00ab	0.06 ± 0.01c	0.06±0.00c	*	0.12±0.00	0.12±0.01	0.12±0.01	0.12 ±0.00	0.11±0.01	ns	***	ns
*Ketones*														
Ethyl isodiacetyl (mg/L)	2.1±0.08c	2.4±0.02ab	2.3±0.03ab	2.5± 0.00a	2.2±0.1 bc	*	2.1±0.08	2.1±0.08	2.2±0.07	2.3±0.09	2.2±0.07	ns	*	ns
*Total other esters*														
Ethyl butyrate (μg/L)	41.7±2.4c	55.0±2.6a	49.9±2.6ab	47.0±0.7bc	47.0±1.1bc	*	41.2±2.1	40.3±4.1	41.9±3.0	43.5 ± 3.3	39.3 ± 2.8	ns	***	ns
Ethyl-2-methylbutyrate (μg/L)	5.8±0.1b	6.9±0.05ab	7.6±0.7a	6.8±0.2ab	6.2±0.3b	*	4.0±0.7	3.3±0.1	3.4±0.2	3.3±0.1	3.4±0.1	ns	***	*
Ethyl isovalerate (μg/L)	7.2±0.1c	9.1±0.2a	9.0±0.2a	8.5±0.1ab	7.9 ± 0.4bc	***	3.7±0.3	3.5±0.2	3.5±0.2	3.6 ±0.1	3.6±0.1	ns	***	***
*Total acids*														
Isobutyric acid (μg/L)	1.4±0.07 b	1.6±0.05b	1.5±0.04b	1.8±0.10a	1.5±0.09b	*	2.1±0.12	2.2±0.06	2.8±0.37	2.7±0.21	2.9±0.34	ns	***	ns
*Total carbonyl compounds*														
Benzaldehyde (μg/L)	0.90 ± 0.2	1.34 ± 0.3	0.60±0.0	1.60±0.5	0.91±0.1	ns	1.06±0.05	1.20±0.08	1.14±0.05	1.17±0.02	1.14±0.02	ns	ns	ns
*Total Terpenes*														
β-Myrcene (μg/L)	0.06±0.01	0.07±0.01	0.06±0.00	0.06±0.02	0.07±0.00	ns	0.08±0.00	0.08±0.01	0.08±0.00	0.09±0.01	0.09±0.00	ns	**	ns
α-Terpinene (μg/L)	0.24±0.01bc	0.21±0.01c	0.21±0.01c	0.27±0.01ab	0.31±0.01a	**	0.14±0.01a	0.11±0.01b	0.11±0.01b	0.12±0.01ab	0.12±0.01ab	.	***	**
cis-Rose-oxide (μg/L)	0.11±0.01c	0.12±0.01abc	0.12±0.02bc	0.12±0.01a	0.15±0.01ab	.	0.07±0.01b	0.08±0.01ab	0.07±0.01ab	0.09±0.01ab	0.09±0.01 a	.	***	ns
Linalool (μg/L)	2.0±0.07b	2.2±0.02a	2.0±0.06b	2.2±0.07a	2.0±0.03b	*	1.8±0.07	1.8±0.01	1.8±0.09	1.8±0.09	1.7±0.02	ns	***	ns
Nerol (μg/L)	0.12±0.01a	0.11±0.01ab	0.09±0.01b	0.09±0.01b	0.12±0.01a	*	0.17±0.01	0.16±0.01	0.16±0.01	0.16±0.01	0.15±0.01	ns	***	*
Nerolidol (μg/L)	2.4±0.05	2.8±0.07	2.4±0.07	2.9±0.23	2.8±0.21	ns	1.63±0.03a	1.48±0.08ab	1.50±0.16ab	1.27±0.02b	1.25±0.03 b	*	***	*
Farnesol (μg/L)	0.81±0.02a	0.73±0.07a	0.47±0.04b	0.68±0.08ab	0.67±0.08ab	*	0.81±0.03	0.60±0.08	0.65±0.11	0.56±0.01	0.57±0.04	ns	ns	ns
*Total norisoprenoids*														
β-Damascenone (μg/L)	3.3±0.01a	3.0±0.17ab	2.8±0.04b	3.0±0.14ab	3.1±0.09ab	.	3.6±0.14a	3.3±0.05ab	3.1±0.08bc	2.9±0.09cd	2.8±0.07d	**	ns	**
β-Ionone (μg/L)	0.070±0.001	0.065±0.001	0.064±0.001	0.067±0.003	0.068±0.002	ns	0.043±0.001	0.040±0.002	0.040±0.002	0.039±0.002	0.041±0.001	ns	***	ns

a Values represent means ± SE (n=3) separated by Duncan’s new multiple range post hoc test (p=0.05). Means separated by different letters are significantly different within each year. Significance or non-significance for shade treatment, year (Y) and interactions between year and shade treatment (Y*S) are indicated by: ‘ns’= not significant; ‘.’ p≤0.1; ‘*’ p≤0.05; ‘**’ p≤ 0.01; ‘***’ p≤0.001.

Acetate esters and fatty acid ethyl esters showed varied effects in wines due to shading in 2020. C0 and D5 had the lowest ethyl acetate concentrations compared to the other shade treatments. Likewise, isoamyl acetate was reduced in C0, D4 and D5 wines compared to D1 and D3 wines. Among the shade film treatments (D1, D3, D4 and D5), ethyl hexanoate and ethyl octanoate concentrations were comparable between D1 and D5 wines and were greater than concentrations found in D3 wines. C0 and D5 wines were indistinguishable in ethyl butyrate, ethyl-2-methylbutyrate and ethyl valerate in 2020, with D1 and D3 wines having the highest concentrations of each these ester compounds. Isobutyric acid increased in D4 in 2020. In 2021, there were no significant impacts of shading on acetate esters, fatty acid ethyl esters, ethyl butyrate, ethyl-2-methylbutyrate or ethyl valerate.

The effect of shade films on various terpenes and norisoprenoids was highly dependent on vintage conditions. Alpha-terpinene was highest in D5 wines but was significantly reduced in D1 and D3 wines in 2020. The D4 wines had the most cis-rose-oxide while C0 wines had the least. Linalool concentrations were reduced in C0, D4 and D5 wines. Among the shaded treatments, nerol concentrations were enhanced in D5 wines in 2020, while there was no effect of shading on nerol concentration in 2021. D5 did not differ from the C0 in nerol concentration in 2020. Farnesol in D3 was reduced in 2020 whereas farnesol concentrations were not affected in 2021 wines. Conversely, nerolidol was unaffected by shade films in 2020, whereas significant decreases in nerolidol concentrations were observed in D4 and D5 wines in 2021. It was observed that β-damascenone were elevated in 2020 in C0 wines, yet differences in β-damascenone concentrations were nonsignificant between shade film treatments. In 2021, only significant differences in β-damascenone concentrations were observed in wines, with C0 wines containing the most β-damascenone and D5 wines containing the least. β-ionone concentrations were not statistically significant between all treatments in 2020 and 2021.

### 3.5 Relationships between chemical parameters, flavonoid composition aromatic profiles

**Figure 2:**
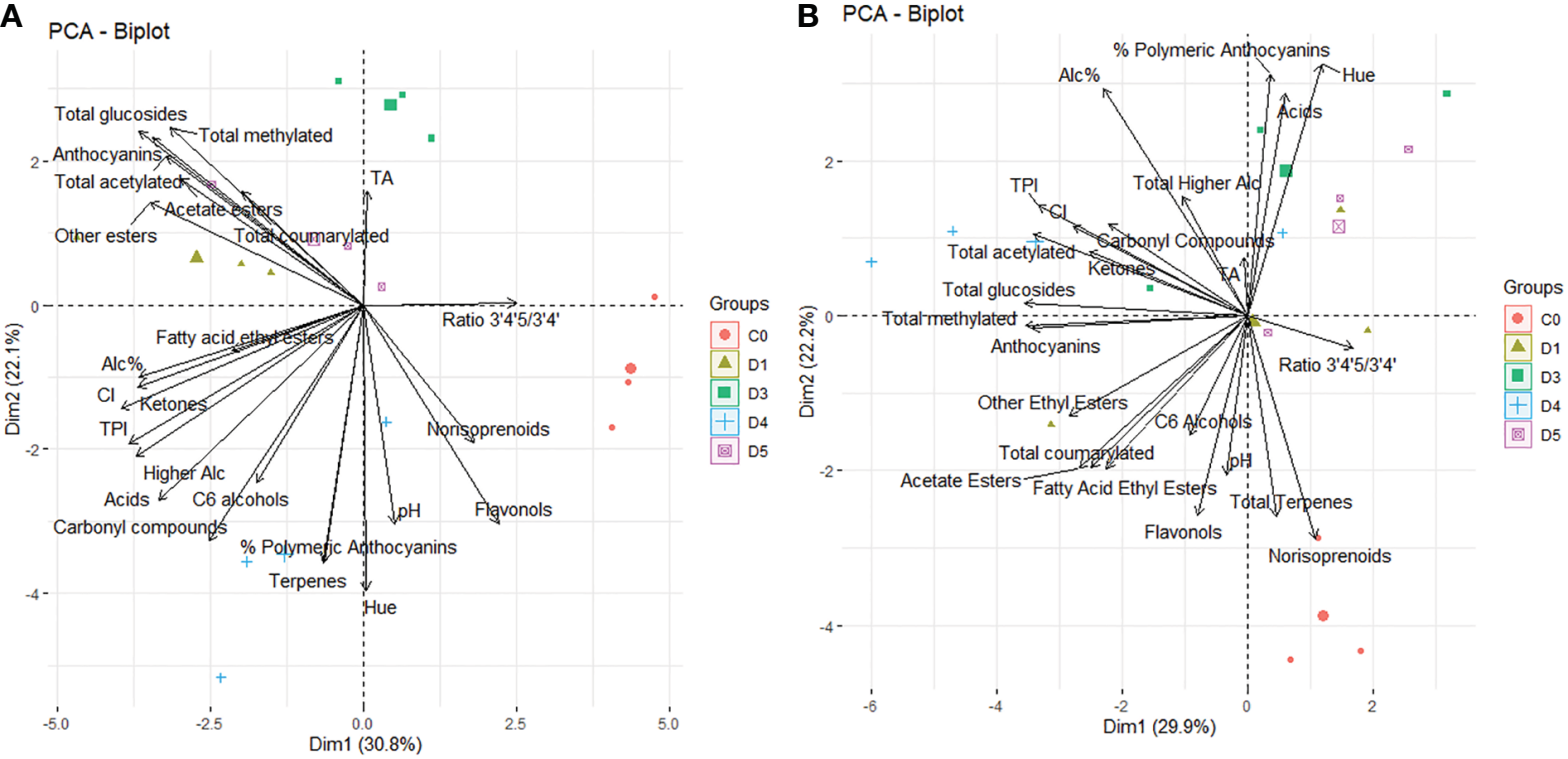
Principal component analysis (PCA) score and loadings plot obtained from the statistical analysis of wine characteristics, flavonoid and aroma profiles of 15 wines from Cabernet Sauvignon grapevines subjected to partial solar radiation exclusion using 4 overhead shade film treatments (D1, D3, D4, D5) and an uncovered control (C0) during the 2020 **(A)** and 2021 **(B)** growing seasons.

To determine the effects of partial solar shading on wine chemistry, flavonoid composition and aromatic profiles of wines we conducted a principal components analysis for both vintages (**Figure 2**). In 2020, PCA indicated that PC1 accounted for 30.8%, and PC2 accounted for 22.1% of the total variance. The C0 treatments clustered together, separately from the partial solar shading treatments. The separation along PC1 was explained by the ratio of di- to tri-hydroxylated anthocyanins in wines, norisoprenoids and flavonols, as well as lower CI, alcohol content and TPI. The separation along PC2 was explained by TA, pH, terpenes and the percentage of polymeric anthocyanins in wine samples. In 2021, PCA indicated PC1 accounted for 29.9%, and PC2 accounted for 22.2% of the total variance. The C0 treatments again separated from shade film treatments, but less so than in 2020. The separation in PC1 was again explained by the ratio of di- to tri-hydroxylated anthocyanins, along with the total glucosides, total methylated anthocyanins and total anthocyanins. The separation of C0 was along PC2 and thus was associated with higher concentrations of flavonols, terpenes, norisoprenoids, and polymeric anthocyanins in wine.

We analyzed the relationships further between the variables monitored with a correlation analysis in wines (**Figure S1**). In 2020, CI in wines had the strongest positive correlation with TPI and acids (**Figure S1**). Alcohol percentage and ketones were also positively correlated to TPI and acids, although less so than CI. Ketones also were very strongly positively correlated with higher alcohols, while higher alcohols were less strongly correlated to acids. Conversely, flavonols were strongly negatively correlated with acetate esters and other esters in wines. Norisporenoids and pH were less negatively correlated to acetate esters. Fatty acid ethyl esters particularly showed to be negatively correlated with TA.

In 2021, the strongest positive correlations in wines were between total anthocyanins and total glucosides and total methylated anthocyanins (**Figure S2**). Total coumarylated anthocyanins were significantly and positively correlated to total anthocyanins, methylated anthocyanins, and total glucosides. Strong negative correlations were found between hue and ester compounds including fatty acid ethyl esters and acetate esters. Alcohol percentage and norisoprenoids were also negatively correlated with each other. A strong negative correlation existed between the ration of di- to tri-hydroxylated anthocyanins and total acetylated anthocyanins. Lastly, total higher alcohols and pH were strongly negatively correlated with each other.

## 4 Discussion

### 4.1 Partial solar radiation effects on wine color and chemical properties were driven by partial solar radiation exclusion

In hot viticulture regions, there is a desire to reduce excessive alcohol content in wines due to marketability and taxation concerns (Varela et al., 2017). Numerous studies have demonstrated that partial solar radiation exclusion is an effective method for reducing the amount of ethanol in wines by reducing TSS in shaded clusters (Joscelyne et al., 2007; Caravia et al., 2016; Lu et al., 2021). However, in the present study, C0 wines consistently had the lowest alcohol content and the lowest concentration of residual sugars in 2020 compared to shaded fruit, despite grapes at harvest having similar TSS values across the treatments (Marigliano et al., 2022). This may be due to the composition of sugars in the grape berry being affected by excessive cluster temperatures in C0 fruit. Sepulveda and Kliewer (1986) showed that heat stress at 40°C post-veraison decreases glucose and fructose in the grape berry. During heat wave events post-veraison, cluster temperatures in C0 reached a maximum temperature of 58°C, exceeding the point at which glucose and fructose content is altered (Marigliano et al., 2022). Additionally, the production of non-fermentable sugars such as arabinose, raffinose and xylose are known to be present in the grape berry (Kliewer, 1965b). Genes involved in the production of these sugars have been shown to be upregulated under heat stress conditions in grapevine (Pillet et al., 2012). While the grape berry is 95-99% glucose and fructose at harvest, these non-fermentable sugars are included in the metric of total soluble solids (Kliewer, 1965a). As a result, while TSS was unaffected by shade films (Marigliano et al., 2022), the proportion of fermentable to nonfermentable sugars may be impacted, thus leading to 2020 C0 wines with reduced alcohol content. This difference in alcohol content between 2021 wines was not observed most likely due to the 2021 growing season being cooler with less GDDs (1572.3°C GDD) than 2020 (1762.7 GDD°C) (Marigliano et al., 2022). While C0 wines in this study demonstrated lower alcohol content than shaded wines, previous literature corroborates cluster temperature reduction by partial solar radiation exclusion as an effective method to lessen sugar content in the grape berry and thus reduce alcohol content of wines (Joscelyne et al., 2007; Caravia et al., 2016; Lu et al., 2021).

The effect of partial solar radiation exclusion in semi-arid climates on berry pH and TA is mixed. Previous work demonstrates partial solar radiation exclusion to reduce pH and increase TA in grape berries by reducing the thermal degradation of organic acids (Martínez-Lüscher et al., 2017; Lu et al., 2021). However, in the present study, berry pH and TA at harvest were unaffected in either year by shade films (Marigliano et al., 2022). Nonetheless, there were apparent effects on wine pH and TA that were vintage dependent. In the present study, D3 wines had the lowest pH and highest TA, while C0 wines did not differ from the shade films D1, D4 or D5 in pH or TA in 2020. Differences observed in pH between the wines ultimately affect the colorimetric properties of these wines. In 2021, D4 and D5 wines showed the highest pH values. It is understood that the pH of the wines can shift the anthocyanin equilibrium in wine solution between the flavylium and quinoidal (colorless) base forms (He et al., 2012a). In the present study, D4 wines had the highest pH and the highest CI. In many cases, when pH rises, CI will decline as anthocyanin equilibrium shifts away from the flavylium form towards the colorless quinoidal forms (He et al., 2012a). However, this was not the case in the present study. Rather, improved color intensity at elevated wine pH could be attributed to co-pigmentation in the wine matrix.

Co-pigmentation refers to non-covalent interactions between anthocyanins and cofactors such as flavonols, flavan-3-ols and proanthocyaninidins, that results in greater absorbance of the wine than color what would be indicated by anthocyanin content and pH conditions (Waterhouse et al., 2016a). Copigmentation in young wines was shown to increase color intensity in young red wines (Jensen et al., 2008). In the hotter 2020 vintage, the total flavonols in grape berries were increased in D4 fruit compared to other treatments (Marigliano et al., 2022). This increased berry flavonol content was transmissible during winemaking, as D4 wines also showed the highest total flavonols with similar concentrations as C0 wines in 2020. TPI was also enhanced in D4 wines. As such, this increased the abundance of cofactors in the wine matrix. Thus, improved color intensity documented in D4 wines in both vintages could be due to the enhancement of absorbance from increased flavonol content by reducing thermal degradation in the vineyard (Marigliano et al., 2022). In the cooler 2021 growing season, shade films produced wines with less flavonols than C0, but greater anthocyanin content, thus leading to improved color intensity in D4 wines.

The increase of phenolic cofactors in D4 wines not only enhanced color and hue, but also led to a higher percentage of polymeric anthocyanins when compared to other shade treatments. Phenolic and polyphenolic compounds from grape skins and seeds can form polymeric pigments in wine with anthocyanins (He et al., 2012b). These polymeric anthocyanins are more stable than monomeric anthocyanins and help to stabilize wine color. This occurs as the proportion of monomeric anthocyanins decreases, leaving color to be maintained by polymeric anthocyanins (He et al., 2012b). Across both vintages, the percentage of polymeric anthocyanins was maximized in D4 wines, indicating that these wines may have greater aging potential than wines from C0 and other shading treatments.

### 4.2 Anthocyanin and flavonol profiles of wine

In the present study, partial solar radiation exclusion modified the composition of anthocyanins in wine. Partial solar radiation exclusion resulted in increased anthocyanin glycosides in wine from shade film treatments except for D4 wines in 2020. In 2021, D4 consistently showed the lowest cluster temperatures post-veraison and as a result, demonstrated the highest concentration of glucosides in resultant wines. Excessive berry temperatures post-veraison in both vintages (>55°C) led to C0 fruit with reduced total anthocyanin content at harvest and this carried over into resultant wines (Marigliano et al., 2022). The reduction of near-infrared radiation by at least 15% produced a cluster temperature conducive to anthocyanin accumulation, as these compounds are susceptible to thermal degradation above 35°C (Spayd et al., 2002). When comparing total anthocyanin and flavonol concentrations between 2020 and 2021, regardless of treatment, 2020 wines had anthocyanin and flavonol concentrations six to seven times less than those in 2021 wines. As flavonoids are susceptible to thermal degradation, this drastic difference in total flavonoid concentrations may be attributed to hotter vintage air temperatures in 2020 compared to 2021.

Previous works show berry sunlight exposure to alter the composition of anthocyanins, such as the proportion of acetylated and coumarylated forms (Haselgrove et al., 2000; Spayd et al., 2002; Downey et al., 2008; Chorti et al., 2010). Modulation of acylated, methylated, and hydroxylated forms of anthocyanins result from the synergistic effect of solar radiation exposure and the coupled increases in berry temperature (Tarara et al., 2008). Generally, high berry temperatures resulting from increased solar exposure results in increased acylated anthocyanins in the grape berry, particularly coumarylated forms (Downey et al., 2008; Tarara et al., 2008). Also, high temperatures result in accumulation of highly methylated anthocyanins such as malvidin derivatives, as these compounds are less likely to degrade than their counterparts (Mori et al., 2007). In 2020, D1 and D5 wines demonstrated highest concentrations of acetylates, coumarylates, and methylated anthocyanins compared to C0 wines. While D1 and D5 treatments demonstrated cluster temperatures less than those from C0 treatments (Marigliano et al., 2022), the concomitant thermal degradation of total anthocyanins in C0 treatments proved to negate any modulation towards acylated or methylated forms in resultant wines. Similarly in 2021, C0, D1 and D5 wines exhibited reduced acylation compared to D4 wines. Again, while D4 consistently exhibited less intense cluster temperatures, the thermal degradation in more exposed treatments eclipsed any identifiable acylation modulation from hot growing conditions. Acylated anthocyanins are more stable compounds and provide color stability and increase blueness in wine (de Rosas et al., 2017; Alappat and Alappat, 2020). However, an increase in methylated anthocyanins will lead to redder hues in wine (Alappat and Alappat, 2020). Therefore, the improvement in acylated and methylated anthocyanin content due to partial solar radiation exclusion may enhance color perception in young red wines through color stabilization and alteration of wine hue.

Likewise, anthocyanin hydroxylation is also directly influenced by temperature and solar radiation exposure. Previous studies on berry exposure utilizing UV selective shade nets as well as leaf removal, demonstrated anthocyanin tri-hydroxylation increases with increasing berry temperature (Chorti et al., 2010). Increases in tri-hydroxylation are driven by accumulation of malvidin derivatives and the temperature sensitivity of F3’H, the catalyzing enzyme for 3’-hydroxylated anthocyanin biosynthesis (Tarara et al., 2008; Chorti et al., 2010). The highest ratio of tri- to di-hydroxylated anthocyanins in 2020 C0 wines were driven by higher concentrations of 3-p-coumaroyl-glucoside derivatives of delphinidin, petunidin and malvidin, despite the ratio of tri- to di-hydroxylated anthocyanins being unaffected at harvest in the grape berry in 2020 (Marigliano et al., 2022). Among shade film treatments in 2020, the reduction of UV light exposure, was the determining factor in anthocyanin hydroxylation patterns rather than berry temperature. Previous shade net studies at the experimental site showed a reduction in UV radiation with black-40% and blue-40% shade nets led to higher anthocyanin tri-hydroxylation in the grape berry compared to control vines at harvest (Martínez-Lüscher et al., 2017). With the reduction of UVB and UVC radiation in D4 and D5 vines, anthocyanin tri-hydroxylation was reduced, regardless of temperature. Ultimately, the upregulation of F3’H from sun exposure could be negated by the reduced catalytic activity of this enzyme under high temperatures experienced in 2020. In the cooler 2021 vintage, the ratio of tri- to di-hydroxylated anthocyanins was unaffected, due to non-significant effect of shade films on acetylated anthocyanins. Ultimately, increased tri-hydroxylation in young red wines will also impact wine hue, resulting in more purple wines (Savoi et al., 2017).

Flavonols in the grape berry skin act as a photoprotectant and are strongly induced by ultraviolet radiation (Agati and Tattini, 2010). Flavonol composition in the grape berry can be used to determine overexposure, specifically by quantifying the molar abundance of kaempferol. C0 berries in this study were shown to be overexposed by surpassing the previously described threshold of approximately 7% molar abundance of kaempferol (Martínez-Lüscher et al., 2019). In both years of the study, flavonol composition in grape berries was maximized in C0 fruit, but D4 and D5 fruit contained the most flavonols across the shade films with minimal thermal degradation of the compounds on the vine (Marigliano et al., 2022). Likewise in both wine vintages, flavonol concentration was modulated by UV radiation exposure, proportional to the amount of UV radiation transmitted to the grapevine. Of the wines produced from shade films treatments, D4 allowed for the most UV transmission while subsequently reducing near infrared transmission by approximately 15%. These light conditions ultimately optimized flavonol content in D4 wines compared to the other shade treatments from both wine vintages. As such, this demonstrated the transmissibility of berry composition under shade treatments to directly improve wine flavonoid profiles. For hot viticulture regions, photoselective solar radiation exclusion provides a strategy to improve not only flavonoid profile but also wine color intensity through copigmentation with anthocyanins.

### 4.2 Wine aroma profiles

C6-alcohols such as 1-hexanol and (*E)*-2-hexen-1-ol are often found in wines as fermentation products. These compounds are derived from microbial mediated cleavage of the C-C double bonds in linoleic and linolenic acids, by lipoxygenase and alcohol dehydrogenate enzymes in yeast (Vilanova et al., 2012; Waterhouse et al., 2016b). Compounds such as 1-hexanol and (*E)*-2-hexen-1-ol are associated with aromas such as cut grass, green, fat and herbaceous aromas and their OAV thresholds are 8000 and 400 ug/L, respectively (Francis and Newton, 2005). The effect of shade films on C6-alcohols was evident in both years; however, there was a yearly effect on which alcohol was altered by the treatment. In 2020, (*E)*-2-hexen-1-ol was the lowest in D4. In 2021, (*E)*-2-hexen-1-ol was unaffected by shade films, while 1-hexanol was highest in C0, D4 and D5. Although there was a statistical difference in C6 alcohols, the differences were not large enough between C0 and treatments to cross the OAV thresholds (Francis and Newton, 2005) for these compounds. Increases of C6-alcohols in C0, D4, and D5 wines may be explained by solar radiation overexposure in the treated clusters. L. He et al. (2020) reported higher linoleic and linolenic acid biosynthesis with leaf removal at veraison. Subsequently, fruit exposed to increased solar radiation had elevated precursors for C6-alcohol production during yeast metabolism. Additionally, L. He et al. (2020) showed higher initial concentration of C6-alcohols in grape berries from leaf removal treatments due to modulation of the volatile compound metabolome and transcriptome in grape berries exposed to sunlight under dry-hot conditions. Therefore, in our experiment which has similar climatic conditions to L. He et al. (2020), fruit from shade films with higher percentages of UV radiation may have both an increase in linoleic and linolenic acids to act as C6-alcohols aromas precursors and increased C6-alcohols in the exposed grape berries. Ultimately, overexposure of the grape berry led to more green and grassy aromas in wine, which may lead to an unripe perception of these wines.

Higher alcohols are also produced during fermentation from yeast metabolism of amino acids. These compounds are generally pleasant aromas including mushroom, roses, honey, candy and fruity notes. Of these compounds, shade treatments increased isoamyl alcohol concentration in 2020 and benzyl alcohol concentration in wines from both vintages. Isoamyl alcohol is associated with solvent and cheese aromas and, while benzyl alcohol is characterized as being citrusy and sweet (Yue et al., 2015). The odor active thresholds for these compounds are 30000 μg/L and 10000 μg/L, respectively (**Table S1**). In 2020, C0 had the lowest concentration of isoamyl alcohol in wines. The effect of shading on the concentration of isoamyl alcohol in wines varies in literature (Lu et al., 2021; Li et al., 2022). In hot growing regions, 75% of total solar radiation exclusion with black polyethylene canopy side shade nets resulted in wines with reduced isoamyl alcohol compared to the uncovered control vines (Lu et al., 2021). However, this experimental site was in a region that received approximately 704.5°C less growing degree days than the present experimental site in the hotter 2020 season, and 514.1°C growing degree days less than the cooler 2021 season. In the study by Lu et al., 2021, reduced solar radiation exposure in a cooler growing region may have resulted in reduced isoamyl alcohol in shaded fruit. When cluster temperatures exceed 42°C in exposed vines, there is a reduction in isoamyl alcohol in resultant wines compared to wines produced from fruit under red and black shade nets (Li et al., 2022). With cluster temperatures of C0 fruit exceeding 42°C, excessive cluster temperatures may be prompting the reduction in isoamyl alcohol and overall wine fruitiness from those produced from overexposed clusters. However, while there was a statistical difference in isoamyl alcohol concentrations between C0 and treatment wines, the effect was not large enough to exceed the OAV threshold for this compound (**Table S1**).

Shade films affected the ester composition predominantly in 2020 wines. Pleasant esters in red wines include ethyl acetate which has a OAV threshold of 12264 µg•L^-1^ and is described as fruity and balsamic (Jiang and Zhang, 2010; Arcari et al., 2017), as well as isoamyl acetate, described as banana aroma with a OAV threshold of 30 µg•L^-1^ (Francis and Newton, 2005). In 2020, ethyl acetate was reduced in C0 and D5 wines, shading and reduced cluster temperatures preserved isoamyl acetate aromas in D1, D3 and D5 wines. When compared to wines from 2021, cooler vintage conditions did not result in ester compositional changes in exposed and shaded wines. Similarly, fatty acid esters were preserved in shaded wines, while 2020 C0 wines consistently had the lowest concentration of all measured fatty acid ethyl esters and various esters, all of which are associated with fruity and candy-like aromas (Jiang and Zhang, 2010). Concentrations of ethyl octanoate and ethyl decanoate remained beneath the reported perception threshold, thus observed shifts in composition with shading may be undetectable in Cabernet Sauvignon wines. However, ethyl hexanoate and ethyl isovalerate have remarkably low OAV thresholds of 5 µg•L^-1^ and 1 µg•L^-1^, respectively (**Table S1**). In the present study, all wines were above these thresholds, indicating that reductions in fruitiness may be perceived. This overall decrease in fruity aromas with cluster exposure and excess temperatures may negatively impact the marketability of Cabernet Sauvignon wines from hot viticulture regions with increasingly more frequent heat wave events associated with climate change.

Unpleasant and rancid aromas include isobutyric acid which imparts a cheese aroma and benzaldehyde which is associated with almond aroma in red wines (Jiang and Zhang, 2010). In this study, isobutyric acid concentrations were only affected in 2020, with D4 having the highest isobutyric acid concentration. The detection threshold for this aroma compound is 2300µg•L^-1^ (**Table S1**). Concentrations detected in the experimental wines were substantially below this threshold, indicating that this slight increase in rancid aromas in D4 wines may not negatively impact overall wine perception. Given that D4 wines also exhibited enhanced fruitiness in with improved ester composition, the trade-off of slight increases in rancid aromas may be offset by the net benefit from increased fruity aromas in the wine aroma profile.

While terpenes are often critical in white wines, these compounds when present in red wines have a large effect on wine aromas as their OAV thresholds are relatively low (Yang et al., 2019). The OAV threshold for a-terpinene, cis-rose-oxide and linalool are 250 µg•L^-1^ , 0.2 µg•L^-1^ and 25.2 µg•L^-1^ , respectively (**Table S1**). The In 2020, α-terpinene, cis-rose-oxide and linalool were all reduced in C0 wines compared D4 and D5 wines, however concentrations of these compounds did not exceed the OAV threshold. These compounds produce odors such as peach, citrus, rose, and floral aromas in red wines (Jiang and Zhang, 2010; Yue et al., 2015). Previous work indicated an increase in terpenoids, particularly linalool in wines produced from fruit under black and red shade nets (Li et al., 2022). It was demonstrated that heat treatment will down-regulate genes encoding key enzymes in terpenoid metabolism in Cabernet Sauvignon grapevines (Lecourieux et al., 2017). Thus, increases in terpenoid content in shade film wines in 2020 may be due to reduced cluster temperature in a growing season with frequent heat wave events. In 2021, C0 wines exhibited the highest concentration α-terpinene, while cis-rose-oxide concentrations remained low in C0, and linalool was unaffected. In 2021, a cooler growing season with fewer days above 38°C may have resulted in less variation in terpenoid composition and net accumulation of terpenoids in exposed fruit (Marigliano et al., 2022). Ultimately, climatic shifts towards more frequent heat wave events will reduce floral and citrus aromas in wines produced from overexposed clusters. However, the year-to-year weather variation will enhance the unpredictability of the development of these compounds, leading to challenges for wine producers looking to produce a consistent product.

As carotenoid breakdown products, C-13 norisoprenoids like β-damascenone often described by sweet and floral aromas (Yue et al., 2015) and has an OAV threshold of 0.05 µg•L^-1^ (**Table S1**). C-13 norisoprenoids have been shown to have a positive linear relationship with sunlight exposure to the grape cluster (Marais et al., 1992; Gerdes et al., 2002; Lee et al., 2007). Under extreme light intensity and temperature conditions, there are decreases in carotenoid concentration in the berry, thus reducing C-13 norisoprenoid precursors. In the present study, β-damascenone was highest in C0, D4 and D5 wines in 2020, while β-damascenone was highest in C0 and D1 wines in 2021, contrary to previous findings in hot viticultural areas. Lee et al. (2007) reported that grape clusters without leaf removal and inner canopy clusters contained more β-damascenone than south-facing clusters exposed to solar radiation by leaf removal. Likewise, black cloth and red shade net enhanced β-damascenone concentration compared to uncovered control (Lu et al., 2021; Li et al., 2022). Despite varied reports, Lee et al. (2007) also demonstrated a linear positive relationship between norisoporenoids in the grape berry and concentrations in wine, with the berry concentration was always greater than that of the resultant wines. It may be possible that carotenoid degradation due to excessive temperatures in C0 treatments was negligible or less than the biosynthesis of C-13 norisoprenoids, resulting in similar concentrations as D4 and D5 shade film treatments. Therefore, the results of this study demonstrated that partial solar radiation exclusion with reductions in UVA, UVB and NIR radiation does not hinder norisoprenoid content in wines. Additionally, the concentrations of β-damascenone across all treatments in both years exceeded the odor active threshold for this compound, indicating that significant differences in β-damascenone concentrations between C0 and treatments may be perceivable in resultant wines.

## 5 Conclusion

Traditional viticulture management practices encouraged solar radiation exposure in wine grape fruit zone to promote flavonoid and aroma development. However, increasingly frequent heat wave events in hot grape growing regions are threating wine quality due to degradation of these desirable compounds in the grape berry (Gambetta and Kurtural, 2021). Thus, to maintain desirable chemical and aromatic properties in red wines, heat mitigation strategies need to be implemented in the vineyard. We determined the cascading effects of partial solar radiation exclusion in the vineyard on resultant wine flavonoid and aroma composition. Overhead shade film D4 produced wines with improved color intensity, TPI, anthocyanin and flavonol profiles compared to C0 wines. Likewise, D4 wines were fruitier and with pleasant aroma profile compared to C0 wines. Ultimately, overhead shade film D4 positively enhanced wine composition in the hotter of the two experimental years, thus demonstrating partial solar radiation exclusion with overhead shade films to be a viable option for maintaining wine quality with forecasted climate change conditions in hot viticulture regions.

## Data availability statement

The raw data supporting the conclusions of this article will be made available by the authors, without undue reservation.

## Author contributions

SK designed the trial. LM, RY, NT, and SK conducted the experiments. AO and CM assisted with volatome analysis, LM curated the data and wrote the first version of the manuscript. All authors contributed to the article and approved the submitted version.

## Supplementary material

Supplementary Table 1 Thresholds of odor active chemicals in young red wines complied by Frank and Newton (2005) from selected recent studies, unless otherwise specified. ^a^ Yue et al., 2015; ^b^ Arcari et al., 2017; ^c^ Jiang and Zhang, 2010; ^d^ Slegers et al., 2015; ^e^ Yang et al., 2019; ^f^ Loscos et al., 2007

Supplementary Figure 1 Correlation matrix among wine characteristics, flavonoid and aroma profiles of 15 wines from Cabernet Sauvignon grapevines subjected to partial solar radiation exclusion using 4 overhead shade film treatments (D1, D3, D4, D5) and an uncovered control (C0) during the 2020 growing season. Circle size and color represent *R* values for Pearson’s correlation analysis. *, **, and *** indicate significance at 5%, 1% and 0.1%, respectively. CI, color intensity; TA, titratable acidity; TPI, total polyphenolic index.

Supplementary Figure 2 Correlation matrix among wine characteristics, flavonoid and aroma profiles of 15 wines from Cabernet Sauvignon grapevines subjected to partial solar radiation exclusion using 4 overhead shade film treatments (D1, D3, D4, D5) and an uncovered control (C0) during the 2021 growing season. Circle size and color represent *R* values for Pearson’s correlation analysis. *, **, and *** indicate significance at 5%, 1% and 0.1%, respectively. CI: color intensity; TA, titratable acidity; TPI, total polyphenolic index.
