# Supplementary material for: Overhead photoselective shade films mitigate effects of climate change by arresting flavonoid and aroma composition degradation in wine
Source: Front Plant Sci. 2023 Jan 27;14:1085939. doi: 10.3389/fpls.2023.1085939 (PMC9912179; doi:10.3389/fpls.2023.1085939)
Supplement: Supplementary Table 1 — Thresholds of odor active chemicals in young red wines complied by Frank and Newton (2005) from selected recent studies, unless otherwise specified. a Yue et al., 2015; b Arcari et al., 2017; c Jiang and Zhang, 2010; d Slegers et al., 2015; e Yang et al., 2019; f Loscos et al., 2007 [file Table_1.docx]

**Table S1. Thresholds of odor active chemicals in young red wines complied by Frank and Newton (2005) from selected recent studies, unless otherwise specified.**

| **Compound** | **Odor Activity Threshold (μg/L)** |
| --- | --- |
| ***Total C6 alcohols*** |  |
| 1-Hexanol (μg/L) | 8000 |
| (*E*)-2-Hexen-1-ol (μg/L) | 400 |
| ***Total higher alcohols*** |  |
| Isoamyl alcohol (mg/L) | 30000 |
| 1-Octen-3-ol (μg/L) | 1^a^ |
| 2-Phenyl-1-ethanol (mg/L) | 10000-14000 |
| Isobutanol (μg/L) | 40000 |
| Benzyl alcohol (μg/L) | 10000^a^ |
| ***Total acetate esters*** |  |
| Ethyl acetate (mg/L) | 12264 |
| Isoamyl acetate (mg/L) | 30 |
| ***Total fatty acid ethyl esters*** |  |
| Ethyl hexanoate (mg/L) | 5-14 |
| Ethyl octanoate (mg/L) | 2-5 |
| Ethyl decanoate (μg/L) | 200 |
| ***Ketones*** |  |
| Ethyl isodiacetyl (mg/L) | 100 |
| ***Total other esters*** |  |
| Ethyl butyrate (μg/L) | 20 |
| Ethyl-2-methylbutyrate (μg/L) | 1-18 |
| Ethyl isovalerate (μg/L) | 1^b^ |
| ***Total acids*** |  |
| Isobutyric acid (μg/L) | 2300 |
| ***Total carbonyl compounds*** |  |
| Benzaldehyde (μg/L) | 2000^c^ |
| ***Total Terpenes*** |  |
| β-Myrcene (μg/L) | 14^d^ |
| α-Terpinene (μg/L) | 250^a^ |
| cis-Rose-oxide (μg/L) | 0.2 |
| Linalool (μg/L) | 25.2 |
| Nerol (μg/L) | 400^d^ |
| Nerolidol (μg/L) | 700^e^ |
| Farnesol (μg/L) | 200^f^ |
| ***Total norisoprenoids*** |  |
| β-Damascenone (μg/L) | 0.05 |
| β-Ionone (μg/L) | 0.09 |

^a^ Yue et al., 2015

^b^ Arcari et al., 2017

^c^ Jiang and Zhang, 2010

^d^ Slegers et al., 2015

^e^ Yang et al., 2019

^f^ Loscos et al., 2007
