# Supplementary figures and images for: Overhead photoselective shade films mitigate effects of climate change by arresting flavonoid and aroma composition degradation in wine
Source: Front Plant Sci. 2023 Jan 27;14:1085939. doi: 10.3389/fpls.2023.1085939 (PMC9912179; doi:10.3389/fpls.2023.1085939)

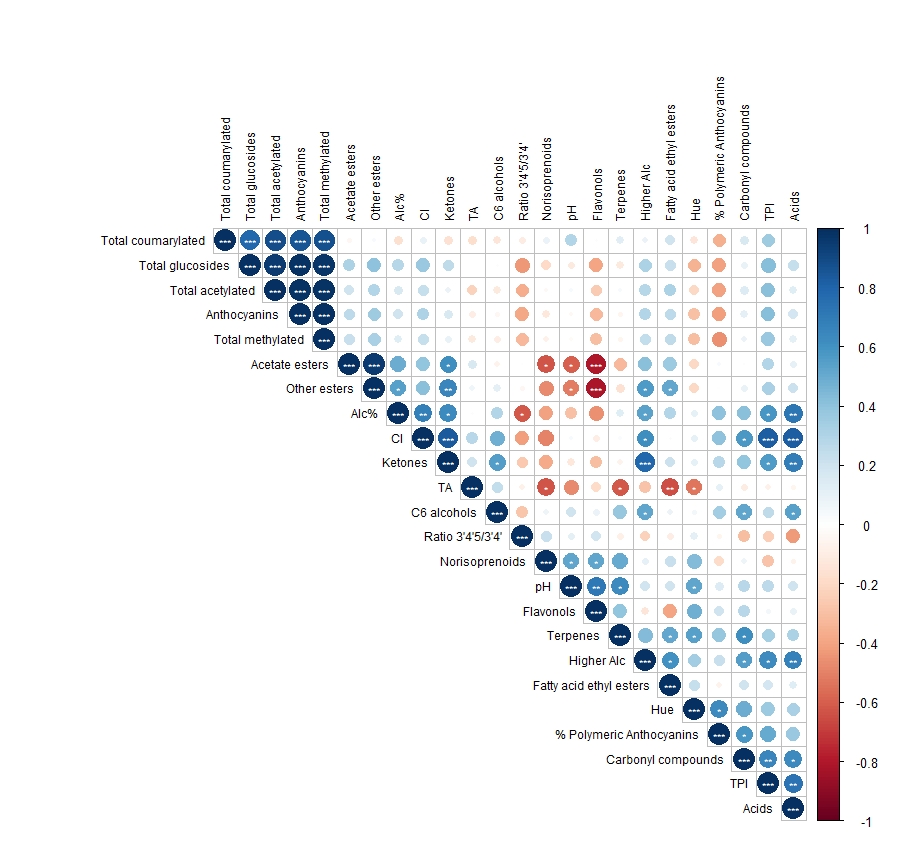

Supplement: Supplementary Figure 1 — Correlation matrix among wine characteristics, flavonoid and aroma profiles of 15 wines from Cabernet Sauvignon grapevines subjected to partial solar radiation exclusion using 4 overhead shade film treatments (D1, D3, D4, D5) and an uncovered control (C0) during the 2020 growing season. Circle size and color represent R values for Pearson’s correlation analysis. *, **, and *** indicate significance at 5%, 1% and 0.1%, respectively. CI, color intensity; TA, titratable acidity; TPI, total polyphenolic index. [file Image_1.jpeg]

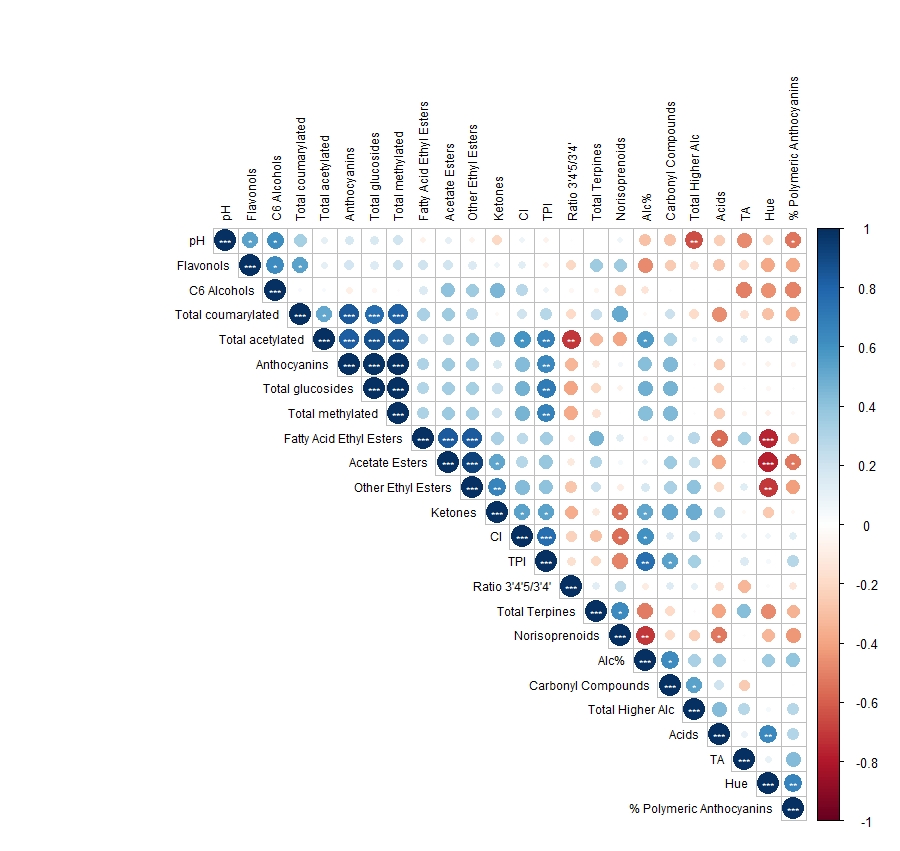

Supplement: Supplementary Figure 2 — Correlation matrix among wine characteristics, flavonoid and aroma profiles of 15 wines from Cabernet Sauvignon grapevines subjected to partial solar radiation exclusion using 4 overhead shade film treatments (D1, D3, D4, D5) and an uncovered control (C0) during the 2021 growing season. Circle size and color represent R values for Pearson’s correlation analysis. *, **, and *** indicate significance at 5%, 1% and 0.1%, respectively. CI: color intensity; TA, titratable acidity; TPI, total polyphenolic index. [file Image_2.jpeg]
